# Profiling of volatile substances by direct thermal desorption gas chromatography high-resolution mass spectrometry for flagging a characterising flavour in cigarette tobacco

**DOI:** 10.1007/s00216-021-03175-0

**Published:** 2021-02-06

**Authors:** Zuzana Zelinkova, Thomas Wenzl

**Affiliations:** European Commission, Joint Research Centre (JRC), Retieseweg 111, 2440 Geel, Belgium

**Keywords:** Cigarettes, Flavour, Volatiles, Mass spectrometry, Tobacco filler

## Abstract

This paper describes an analytical method that supports the implementation of articles 9 and 10 of the WHO Framework Convention on Tobacco Control (FCTC) regarding the provisions on the reduction of the palatability and attractiveness of tobacco products regarding flavour ingredients. This study aimed to develop a screening method to identify cigarettes that may have a characterising flavour to support the implementation of the ban of characterising flavours of tobacco products, as laid down in the US and EU law. An analytical method combining direct thermal desorption and GC–QTOF MS was developed for acquiring the profile of volatile and semi-volatile substances in tobacco. A database of flavour additives was created comprising 133 compounds. A group of cigarettes without a declared characterising flavour was used to establish a reference profile of flavouring chemicals commonly present in tobacco products. A reference profile was modelled both by the means of principal component analysis (PCA) and based on the calculation of threshold values specified as 95th percentile of measured compounds’ relative responses. Cigarettes and roll-your-own tobacco labelled as flavoured were analysed to evaluate the discrimination power of the method. A constructed model of the reference cigarettes allowed the differentiation of the flavoured tobacco products from the reference group. The method allows drawing conclusions on the chemical profiles of flavour constituents of tobacco products at even sensorial subliminal concentration levels and is suitable for both the initial screening of products on the market for characterising flavours and for confirmatory purposes after sensory analysis.

**Supplementary Information:**

The online version contains supplementary material available at 10.1007/s00216-021-03175-0.

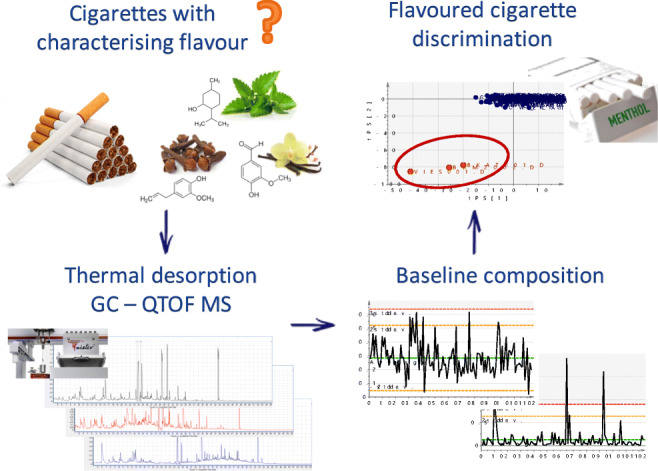

## Introduction

Tobacco additives serve different purposes. They act as humectants, restore the level of carbohydrates, or provide a certain flavour to the cigarette [1–3]. As such, tobacco additives influence the attractiveness of the product to the consumer [4–6]. Several sources reported the effect of flavour attributes of cigarettes on the consumption behaviour of different gender and age groups [4, 6–9]. It was shown that young people prefer flavoured tobacco products carrying a menthol flavour or a sweet caramel flavour, the latter resulting from the combustion of sugars [10, 11]. Menthol is the most widespread example of such flavours [12]. It masks the aroma and taste of cigarette smoke and facilitates the inhalation of smoke due to a “cooling” effect [13]. In its attempt to reduce the attractiveness of smoking, the Conference of the Parties (COP) to the Framework Convention on Tobacco Control (FCTC) recommended in its fourth session to prohibit or restrict ingredients, such as flavours, that facilitate the palatability of tobacco products [14]. Several jurisdictions have transposed this already into legislation [15, 16].

The European Union defines a characterising flavour as “a clearly noticeable smell or taste other than tobacco, resulting from an additive or a combination of additives, including, but not limited to fruit, spice, herbs, alcohol, candy, menthol or vanilla, which is noticeable before or during the consumption of the tobacco product” [15]. Following recommendations from the HETOC consortium [17, 18], EU legislation requires sensorial assessment of unburnt tobacco by an expert panel complemented by chemical analysis for identifying cigarettes and roll-your-own tobacco products carrying a characterising flavour [17]. The sensory assessment of tobacco products has a long history with respect to product design and consumer preference evaluation [8]. The few approaches proposed in literature for the sensory assessment of tobacco products’ potentially exerting characterising flavours differ in the composition of panels, design of experiments, and statistical data evaluation [19–21]. The definition of characterising flavours provides conceptual challenges for both sensorial and chemical analysis [22]. Instrumental analysis of flavour chemicals contained in tobacco might be more sensitive than sensorial analysis; however, the sole presence of a flavour chemical does not necessarily constitute a characterising flavour [23]. A point of reference is needed for the interpretation of both sensory and chemical analysis data regarding the presence of a characterising flavour. Many flavour chemicals are contained in raw tobacco or are generated during processing. Seasonal and batch variability influence their composition and contents. Therefore, a single tobacco product will not suffice as reference [18]. Both chemical and sensorial analysis methods suffer usually from the necessity to define and validate the method ex ante for a set of target analytes or flavour attributes, excluding thereby potential flavours that are relevant for the particular tobacco product being assessed. Chemical analysis has also to deal with interferences caused by the tobacco matrix and potential bias caused by discrimination of flavour chemicals during the analysis [24].

Headspace extraction hyphenated to gas chromatography mass spectrometry is proposed for the characterisation of the volatile fraction of tobacco [18, 25, 26]. An increased level of sensitivity provides headspace extraction methods combined with a pre-concentration step such as solid-phase micro-extraction (SPME) [22, 27–29]. Gas chromatography–tandem mass spectrometry was employed for increasing selectivity compared to single quadrupole mass spectrometry in the analysis of 23 flavour additives in tobacco extracts [30].

Taking account of the above-mentioned conditions and limitations, the current study proposes a chemical analysis method which is complementary to sensorial analysis and which may be used for screening or confirmatory purposes. Direct thermal desorption, which showed earlier little discrimination in the analysis of tobacco smoke constituents, was applied for the extraction of volatile and semi-volatile substances from tobacco filler (TF) [31]. Gas chromatography QTOF mass spectrometry (GC–QTOF MS) allowed simultaneously the selective and sensitive measurement of target flavour chemicals and recording of non-target signals. In total, 126 different cigarettes without a declared characterising flavour (WDCF) were used for creating a reference chemical profile of flavour additives and for establishing a discrimination model. The developed model was scrutinised by the measurement of commercial tobacco products having a characterising flavour.

## Materials and methods

### Samples

One hundred twenty-six WDCF cigarette samples of different brands were randomly collected at licenced tobacconists in 22 European countries. Cigarette brands and country of purchase are given in Table S1 (see Supplementary Information, ESM).

Additionally, 60 samples of flavoured cigarettes and flavoured roll-your-own tobacco were acquired predominantly outside of Europe. Details on the flavoured tobacco products are summarised in Table S2 (see ESM).

The research cigarette 3R4F obtained from the University of Kentucky was used for quality control (Lexington, KY, US).

### Chemicals

Isotopically labelled 2-ethylphenol-D_10_ was obtained from CDN isotopes (Quebec, Canada). Methanol of LC-MS grade was purchased from VWR (Leuven, Belgium). The spiking solution of isotopically labelled standard in methanol was prepared gravimetrically from an initially prepared stock solution (1.5 mg/mL).

### Equipment and instrumentation

The direct thermal desorption GC–QTOF MS system consisted of a thermal desorption unit (TDU, Gerstel, Mülheim an der Ruhr, Germany), operating automatically in conjunction with a MultiPurpose Sampler (MPS, Gerstel), a gas chromatograph 7890A (GC, Agilent Technologies, Santa Clara, CA, USA) equipped with a cooled injection system (CIS) and a programmable temperature vaporizing inlet (PTV, Gerstel), and an Agilent 7200 Accurate Mass QTOF mass spectrometer (Agilent Technologies).

### Analysis method

For the preparation of a test sample, three cigarettes were randomly selected from the cigarette package. The TF was separated from the cigarette paper and cigarette filter, ground, and homogenised in a mortar under cooling by liquid nitrogen. A volume of 5 μL of isotopically labelled standard solution (30 μg/mL in methanol) was pipetted into a glass micro-vial insert, and a portion of 30 mg of sample was weighted over the standard. The micro-vial insert was inserted into a glass thermal desorption tube and placed on the autosampler for analysis.

Thermal desorption was realised in splitless mode by ramping the TDU from 20 °C held for 0.1 min to 100 °C at 30 °C/min and holding for 15 min with a helium purge flow of 100 mL/min. Volatile components were trapped in the PTV inlet on a commercial liner containing Tenax TA (Gerstel) at a temperature of 15 °C. The trapped compounds were transferred onto the HP-5MS GC column (30 m × 250 μm × 0.25 μm, Agilent Technologies) in split mode, with a spit ratio of 15:1, while programming the PTV inlet from 15 °C held for 0.8 min to 270 °C at 12 °C/s held for 30 min. The GC oven was programmed from 45 °C (held for 2 min) to 210 °C at 4 °C/min and to 300 °C at 10 °C/min (held for 5 min). Helium was used as a carrier gas at a constant flow rate of 1 mL/min. The transfer line was set to 300 °C. The QTOF MS was operated in EI mode at 70 eV ionisation energy. The ionisation energy was reduced to 25 eV for the time window from 23.25 to 23.95 min, when nicotine eluted, in order to reduce ionisation of nicotine and to avoid saturation of the MS detector. The data acquisition rate was 5 Hz in extended dynamic range (EDR) mode for the mass range of *m/z* 45–450. Mass resolution was about 6500 at *m/z* 219.

The quality control sample (3R4F research cigarette) was measured with each sample batch together with two blank samples (empty desorption tube).

Some of the flavoured cigarette samples contained the flavour in a capsule in the filter. The capsules were broken, and the cigarettes equilibrated with the flavour for 24 h in a closed vial. The tobacco of these cigarettes was analysed after this period in the same manner as other tobacco samples. However, it has to be noted that the applied process does not reflect real use conditions.

### Dilution experiments

Tobacco from ten randomly selected samples from the group of WDCF cigarettes were ground and homogenised by means of a T2F laboratory Turbula mixer (WAB, Muttenz, Switzerland). The prepared mixture was used for dilution of TFs from five flavoured cigarettes in the ratio of 1:1 and 1:5. Each sample of diluted flavoured tobacco was homogenised and analysed applying the method described above.

### Compound identification and data analysis

Chromatograms were first subjected to deconvolution and automatic peak detection. Substances detected in the TFs of WDCF cigarettes were identified based on the comparison of acquired mass spectra with reference spectra in the NIST library and based on the comparison of their linear retention indexes with those reported in the literature [32, 33]. The library match factor above 940 was considered for the compound identification. Next to the quantifier ion, two qualifier ions were used for compound identification. An in-house flavour database was built comprising compounds, which are listed in the Leffingwell flavour database as flavour additives in TF [34]. Additionally, flavour compounds used in liquids for electronic cigarettes were included in the database.

An integration method was developed using the MassHunter Quantitative analysis. The response (area under the peak) of the respective compound relative to 2-ethylphenol-D_10_ was used for further data processing. In case a compound included in the in-house database was not detected in a sample, the signal abundance was set to one (1) for data evaluation purposes.

Principal component analysis was performed after data pre-processing by mean-centering and scaling to unit-variance using the statistical software SIMCA.

### Software

MassHunter Software of Qualitative Analysis version B.07.00 (Agilent Technologies), Quantitative Analysis for TOF version B.07.01 (Agilent Technologies), and PCDL Manager version B.07.00 (Agilent Technologies) were used for data analysis. Mass spectra were compared with the NIST mass spectral library version 2.0 2011 (NIST, Gaithersburg, MD). The general flavour description of the compounds and information on their use as tobacco additives was extracted from the Leffingwell Flavor-Base (10th edition, Leffingwell & Associates, Canton, GA, USA). Multivariate data analysis was carried out by using SIMCA version 15.0.2. (MKS Umetrics, Malmo, Sweden).

## Results

### Flavouring database

The analytical method focused on volatile and semi-volatile components of TF. Examples of the total ion chromatograms are given in Fig. S1 (see ESM). The acquired chromatograms consist of approximately 200 substances in each sample. These substances represent both the compounds that are naturally present in processed tobacco and added compounds. Only the compounds known as cigarette flavour additives [34] were selected for building the in-house database. Additionally, pre-existing information on the composition of vanilla, mint, strawberry, cherry, and other flavoured liquids for e-cigarettes was incorporated into the in-house database, as similar flavour formulations might be used in flavoured cigarettes. The aggregated database comprised 133 compounds (ESM Table S3). The list of flavour compounds is not exhaustive, but was assumed sufficient for demonstrating the power of chemical analysis in discriminating cigarettes with characterising flavour from the group of WDCF cigarettes.

The relative responses of the compounds were examined for their distribution pattern. In general, two types of distribution pattern were detected. Bimodal distributions were observed for compounds, which are both naturally present in tobacco, and used as additives. Skewed distributions were observed more frequently, comprising compounds solely used as additives. Twenty-five compounds were not detected in the WDCF cigarette group. These compounds represent a group of flavourings, which might be potentially added to flavoured cigarettes. Examples of data distribution patterns are shown in Fig. S2 (see ESM).

### Principal component analysis

**Fig. 1:**
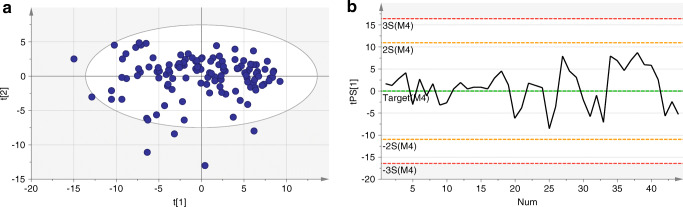
**a** PCA score plot of PC1 and PC2 of the developed model for the group of reference WDCF cigarettes. PC1 and PC2 captured 29.3% of total variance. **b** Control (Shewhart) chart of the quality control sample of the PC1 score. Green line represents variable average of the model. The warning (yellow lines) and control (red lines) limits are set as 2 and 3 times standard deviations from the average, respectively

A multivariate statistical model based on principal component analysis (PCA) was established for the identification of TF having a characterising flavour. The 126 WDCF cigarettes were used as a reference for building the model. These cigarettes formed the reference space as denoted elsewhere [18]. The Hotelling’s T2 range plot calculated for three principal components’ model indicates the distance from the model space for each observation and was used for the investigation of outliers in the data set. Three cigarette samples were positioned far above the critical limit (95% confidence level), which indicates that they were far away from the other observations in the score space (ESM Fig. S3). The three samples contained between 8 and 23 scaled and mean-centred scores (representing compounds such as methypyrazine, dimethylpyrazine, guaiacol, ethylguaiacol, citronellol, γ-decalactone, and γ-undecalactone p-anisyl acetate) at intensities at minimum three standard deviations higher than the average scaled and mean-centred scores of the PCA model. These samples were regarded as strongly flavoured products and were excluded from the PCA model for precautionary reasons. A new model was established comprising 123 reference WDCF cigarettes. The model was not further refined to avoid overfitting. The final PCA model consisted of three principal components and captured 33.6% of the total variance. The cumulative variation predicted by the model (cumulative Q^2^) was 21.1%. The PCA score plot for PC1 and PC2 is presented in Fig. 1a. A multivariate control chart was set up for the 3R4F quality control sample in order to keep the performance of the method under statistical control (Fig. 1b).

### Screening of tobacco products for the potential presence of a characterising flavour

**Fig. 2:**
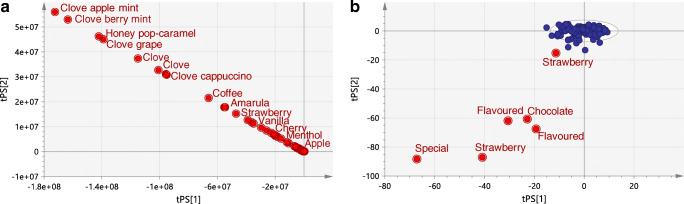
PCA score plot of PC1 and PC2 of the reference model with the flavoured tobacco products projected in the principal components space (**a**) and the zoomed plot with the six flavoured samples positioned closest to the reference group (**b**). Red dots represent the flavoured products; blue dots represent the reference group of WDCF cigarettes

**Fig. 3:**
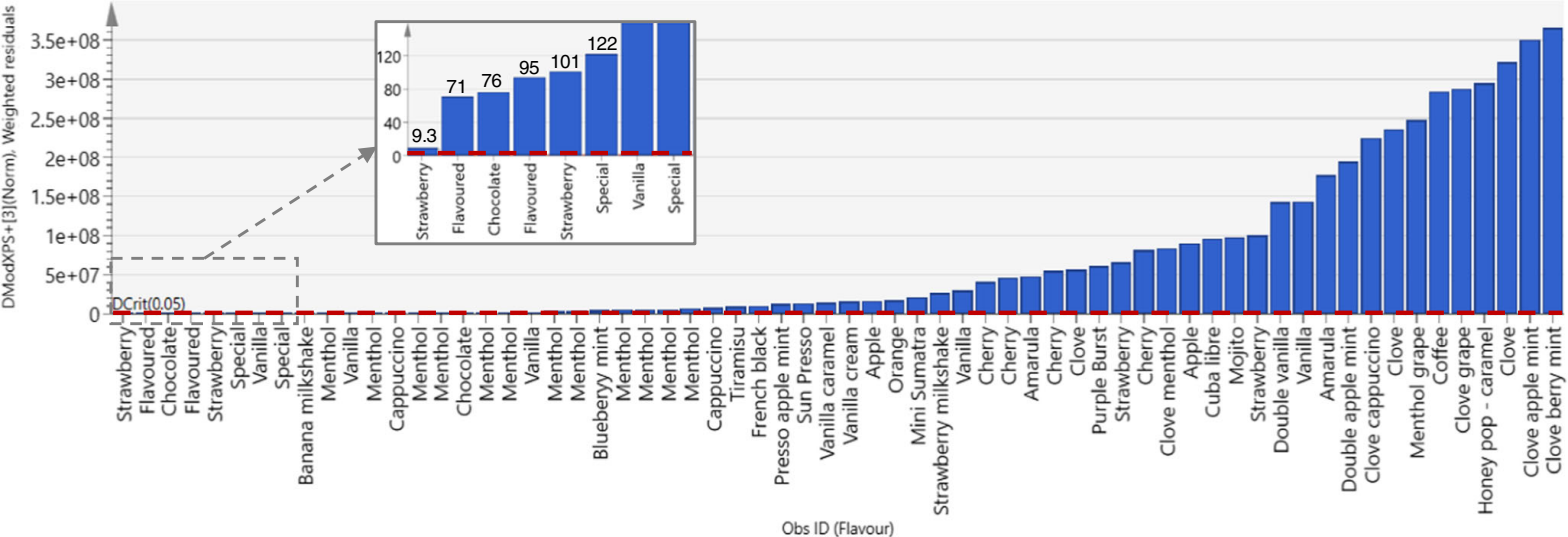
DModX PS+ plot of a distance to the model observed for the flavoured tobacco products. Plot is in normalised unit (absolute distance divided by the pooled residual standard deviation of the model). The red dashed line represents the critical value (DCrit), equal to 1.18. The left part of the plot is magnified to show the samples with the lowest distance values

Relative responses of the target components measured in the flavoured tobacco samples were projected into the PCA model of reference WDCF cigarettes. The majority of the flavoured cigarettes was positioned so far away from the reference space that in the score plot, the reference group around the origin of PC1 and PC2 is no longer visible (Fig. 2a). Figure 2b displays the rescaled PCA score plot with the six flavoured samples closest to the reference group. A strawberry-flavoured cigarette was positioned closest to the reference group, but still outside the 95% tolerance ellipse. The distance to model (DModX PS+) plot was used for the evaluation of the flavoured tobacco products. Distance to model is an estimate of how far the observation is positioned in space from the model plane. The critical limit for the distance to model (DCrit) is computed from the F-distribution function of the reference group at a significance level of 0.05 and has a value of 1.18 for the reference model. A DModX PS+ value twice as high as the critical distance indicates that the observation is statistically significantly different from observations in the reference model. The tested flavoured tobacco products were far above the critical value as shown in Fig. 3. Even the strawberry-flavoured cigarette located closest to the reference group in the PCA score plot was characterised by a DModX PS+ value of 9.3.

**Fig. 4:**
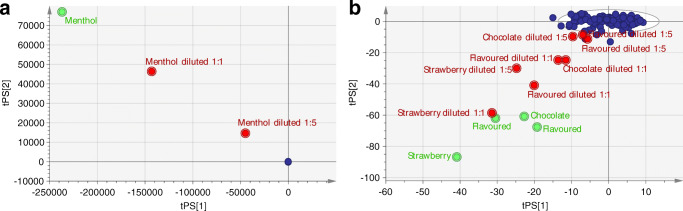
PCA score plot of PC1 and PC2 of the reference model with projected diluted flavoured tobacco products in the principal components space. Blue dots represent the reference group of WDCF cigarettes, green dots the original flavoured samples, and red dots the diluted flavoured samples. First plot (**a**) visualises only the menthol cigarette and its dilution; second rescaled plot (**b**) displays the dilution of other four flavoured samples

Four flavoured TFs, located close to the reference group, and one menthol-flavoured TF were diluted in a ratio of 1:1 and 1:5 with a homogenised mix of tobacco filler from WDCF cigarettes. Diluted samples were still clearly discriminated by the PCA model, although three test samples diluted in the ratio 1:5 were located close to the 95% tolerance ellipse (Fig. 4). However, the calculated distance to the model was above the DCrit value (1.18) for all of the diluted samples starting with 4.4 for “flavoured cigarette” diluted 1:5 (ESM Fig. S4).

### Flavour profiles

**Fig. 5:**
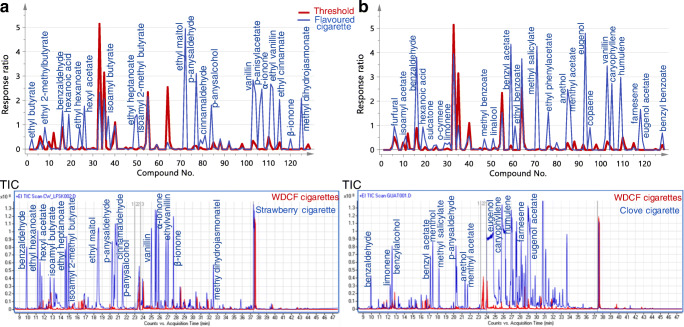
Upper: Overlay of the profile of threshold values (red line) with the profile of the relative responses measured in the **a** strawberry-flavoured cigarette and **b** clove cigarette (blue lines). Lower: Overlaid total ion chromatograms (TIC) of tobacco fillers of six reference WDCF cigarettes (red lines) and tobacco fillers of **a** strawberry-flavoured cigarette and **b** clove cigarette (blue lines)

Additionally to PCA, the flavour profiles of the flavoured products were investigated and compared to threshold values derived for each of the 133 target substances from the reference WDCF cigarettes. Due to the absence of odour threshold values for flavour substances in tobacco, arbitrary threshold values were established as 95th percentiles of the relative abundances measured in the reference group of 126 WDCF cigarettes. As PCA already indicted “outliers”, the exclusion of the highest relative abundances aimed to prevent any unintentional blurring of the baseline profiles by flavoured samples. It has to be noted that these threshold values are just indicative and serve only to identify compounds with high abundances in TF samples flagged by PCA. The profile of the flavour components in flavoured products was then plotted against the threshold values. Examples of overlays of the profile of threshold values and relative responses measured for selected flavoured cigarettes are shown in Fig. 5 together with the respective overlaid total ion chromatograms. As visualised, the amounts of flavouring substances extracted from the products were substantial, leading partially to saturation of the MS detector. In the depicted examples of strawberry-flavoured and clove (also known as “kretek”) cigarettes, 31 and 40 components were at least twice as abundant as the threshold value. High contents of compounds with characteristic fruity flavours were detected in the strawberry cigarette, such as ethyl butyrate, ethyl 2-methyl butyrate, isoamyl 2-methylbutyrate, isoamyl butyrate, ethyl hexanoate, and ethyl heptanoate as well as sweet flavours including ethyl maltol, vanillin, and ethyl vanillin. The clove cigarette contained significant amounts of typical compounds, such as eugenol, eugenol acetate, caryophyllene, and copaene, compounds providing a spicy flavour. More examples of the overlaid profiles and total ion chromatograms can be found in Fig. S5 (see ESM).

## Discussion

Under the EU law, the judgment whether a tobacco product has a characterising flavour requires a comprehensive testing strategy, which comprises both sensorial and chemical testing, as sensorial testing is unlikely to identify the source of the flavour and chemical analysis cannot draw firm conclusions on the “clearly noticeable” character of flavour chemicals or the overall sensorial impression [17, 18]. Due to the lack of a widely recognised numerical description of the flavour profile of cigarette tobacco, the establishment of baseline levels of flavour chemicals contained in WDCF cigarettes on the European market was required as a basis for developing classification model. It was assumed that a significantly large range of cigarettes would depict the variability of volatiles contained in the tobacco products at the time of sampling. Sampling did not take into account market share; the collected cigarettes represent, however, more than 10% of cigarette brands on the EU market [35]. Cigarettes with label declaring characterising flavour, except menthol flavour, were already removed from the EU market at the time of sampling. Menthol cigarettes were excluded from sampling. Therefore, it was not expected to sample, if at all, many products with a characterising flavour. This justified the initial assumption that these reference cigarettes were free from any characterising flavour. However, the reliability of the assumption was scrutinised in a first iteration of data analysis and three “strange” products were excluded from the reference group before performing a second iteration of data analysis on the reduced set of reference products.

The experimental approach comprised the establishment of a chemical baseline profile of flavour components present in TF of WDCF cigarettes, and comparing profiles of suspect products to the baseline profile.

Presuming adaptation to the local situation, the presented analytical method could also support the implementation of the ban of characterising flavours in other jurisdictions, such as the USA. It provides a comprehensive profile of volatile and semi-volatile components present in unburned TF. It is less prone to discrimination than comparable SPME-based methods, which experience competition effects on the fibre and pH-dependent extraction efficiencies [24]. However, it is susceptible to saturation of the detector, as observed in case of flavoured tobacco products. The overloading can be reduced by lowering the amount of sample introduced into the system. However, this will negatively affect the sensitivity of the method for less abundant compounds. High-resolution full scan mass spectrometry supports peak deconvolution and identification of chemically low abundant, but potentially sensorial relevant substances.

Two routes were taken for discriminating profiles of flavour compounds contained in flavoured TF from the baseline profile. The first approach used a statistical model employing PCA. A confidence limit was derived for the reference group, followed by the projection of potentially flavoured test samples into the principal components’ space. The classification of a test sample is achieved by comparing its residual variance to the residual variance of the reference group. The critical limit for the distance to the model is based on the F-distribution (95% confidence level). The developed PCA model consisting of three principal components captured 33.6% of the total variance. The relatively low percentage can be explained by the high variability of the flavour components in the reference group and the inclusion of targeted flavour compounds in the model, which were not detected in any reference WDCF cigarette. A large distance to the reference group was observed when projecting the flavoured TFs into the model. The model correctly identified all the flavoured products as not belonging to the group of reference cigarettes, which was due to the high abundances of flavour substances recorded in chromatograms of flavoured TF.

Krüsemann et al. [23] demonstrated that the sensorial threshold value for a menthol-flavoured TF (~ 1.8 mg/g) was about 1000 times higher than the odour threshold of (-)-menthol in water. Even so, the question was raised whether the analytical method would be still able to discriminate flavoured from WDCF cigarettes if the amount of flavourings would be less abundant. For that reason, five selected flavoured TFs were diluted in ratios of 1:1 and 1:5 with non-flavoured TF. The diluted samples could be still discriminated from the reference group, which highlights the power or the model.

The PCA-based approach does not provide information on the composition of a characterising flavour. Comparison of the threshold values for WDCF cigarettes (specified as 95th percentile of relative responses of products in the reference group) with the profile of the flavour components allows identifying flavouring substances that are likely responsible for the characterising flavour. All tested flavoured tobacco products contained a considerable amount of flavourings, most likely to outweigh the intense tobacco flavour, which lessens the probability that the proposed approach will “overlook” the presence of additives being responsible for eliciting a characterising flavour.

## Conclusions

An analysis method was developed for flagging tobacco filler that potentially exert a characterising flavour to support the implementation of Directive 2014/40/EU. This method takes into consideration the high variability of flavour additives usually present in tobacco products. It is based on the comparison of the profile of 133 flavour compounds in products without a declared characterising flavour (reference products) with the profiles recorded for the investigated samples. The analysis of selected flavoured tobacco products showed that these products contain remarkably high amounts of flavouring substances, which were clearly discriminated from the reference products.

The developed method represents a valuable tool for tobacco control. It can be applied for screening tobacco products on the market to identify those potentially carrying a characterising flavour, which might be further tested by a sensory panel. Additionally, the method can be used for confirmation purposes, complementary to sensory analysis as defined in the Commission Implementing Regulation (EU) 2016/786 to prove that a clearly noticeable characterising flavour is indeed caused by additives.

## Supplementary information

ESM 1 (PDF 0.99 mb)

## Data Availability

All data generated or analysed during this study are included in this published article and its supplementary information files.
