# Supplementary material for: Profiling of volatile substances by direct thermal desorption gas chromatography high-resolution mass spectrometry for flagging a characterising flavour in cigarette tobacco
Source: Anal Bioanal Chem. 2021 Feb 6;413(8):2103–11. doi: 10.1007/s00216-021-03175-0 (PMC7943432; doi:10.1007/s00216-021-03175-0)
Supplement: Supplementary file 1 — (PDF 0.99 mb) [file 216_2021_3175_MOESM1_ESM.pdf]

Analytical and Bioanalytical Chemistry

Electronic Supplementary Material

Profiling of volatile substances by direct thermal desorption gas chromatography high-resolution mass spectrometry for flagging a characterising flavour in cigarette tobacco

Zuzana Zelinkova, Thomas Wenzl

European Commission, Joint Research Centre (JRC), Geel, Belgium

Thomas.Wenzl@ec.europa.eu

Table S1 Brands, producers and country of purchase of cigarettes without declared characterising flavour (WDCF) which were included in the reference group

Brand Name	Producer	Country	Brand Name	Producer	Country	Brand Name	Producer	Country
Afri	Landewyk Tobacco	DE	Falk Classic	JTI	AT	Overstolz*	JTI	DE
Absolut	Greenleaf Tobacco	GR	Fortuna	Altadis/IT	ES	Pall Mall Red	BAT	CZ
Allure	Von Eicken	DE	Fred Red	Fred & Fly	DE	Parisienne Jeune	BAT	DE
American Legend	KTC Inc.	ES	Free Jack	Heupink & Bloemen	FR	Parker & Simpson Red	IT	FI
American Spirit	JTI	AT	Gauloises Blue	IT	CZ	Parliament Blue	PMI	DE
Austin	Landewyk Tobacco	BE	Giants Red	ALDI	DE	Pepe	Von Eicken	DE
Bacco Silverline	Bacco Tobacco	AT	Gitanes Filtre	Altadis/IT	FR	Petra	PMI	SK
Bastos	IT	BE	Gladstone Classic	BAT	NL	Philip Morris Blue	PMI	CH
Batton	Von Eicken	AT	Glamm	JTI	NL	Pine Blue	KT&G	GR
Belinda	BAT	NL	Gold Mount	Moda Palace	GR	Pink Corset	KT International	FR
Benson & Hedges Red	Gallaher Group - JTI	IE	Golden Gate	IT	DK	Pool Red	Heupink & Bloemen	FR
Blend	JTI	SE	Golden Smart	JTI	AT	Portugues	Tabaqueira	PL
Bonus Red	La Suerte Cigar & Cigarette Factory	FI	Goldfield Red	Grand River Enterprises	BE	President Slims	Papastratos International	GR
Boston Silver	Western Tobacco Corp.	BE	Hobby	JTI	AT	Prince	BAT	DE
Boule d'Or	BAT	BE	Houston	Western Tobacco Corp.	BE	Pueblo	Pöschl Tabak	DE
Break Blue	Scandinavian Tobacco Group	NL	John Player Special	IT	ES	Regal	IT	UK
Brera Slim	BAT	IT	Karelia Afoi	Karelia	GR	Regina Red	CTIEC	RO
Brooklyn Red	Brooklyn Tobacco Factory	FR	Kent Surround	BAT	BE	Reval	IT	DE
Buffalo	Six Nations Manufacturing	DE	Kingdom	The Dominion Tobacco Co. Ltd	GR	Reyno White	JTI	DE
Bulgartabac Special	Bulgartabac Holding Group	BG	L&M Red	PMI	FI	RGD	PMI	GR
Burton	Von Eicken	DE	Lampsi	European Tobacco	GR	Rone Slim	Grand River Enterprises	GR
Camel	JTI	PT	Lex	Landewyck Tobacco	NL	Roth Handle	IT	DE

Capital	Capital Tobacco Ltd.	GR	Lexington	Landewyck Tobacco	NL	Rothmans Red	BAT	IT
Casablanca	JTI	AT	Lido Red	BAT	IT	Royal Blue	IT	GR
Che	Landewyk Tobacco	AT	Liggett Ducat	JTI	PL	Royale Red	IT	FR
Chesterfield Red	PMI	PT	Lord Extra	BAT	DE	Silk Cut	Gallaher Group - JTI	ES
Colorado	Western Tobacco Corp.	BE	Lucky Strike Red	BAT	SK	Smart	Gallaher Group - JTI	AT
Coronas	JTI	ES	MAC White	BMJ Industries	GR	Sopianae Blue	BAT	HU
Cpedeli	Bulgartabac	BG	Manitou	Von Eicken	DE	Sparta	PMI	CZ
D&B Comfort	CTIEC	RO	Marlboro Red	PMI	SK	Stuyvesant Red	BAT	IT
Dames	JTI	AT	Mayfair	Gallaher Group - JTI	IE	The King	KT International	BG
Davidoff	IT	SE	Meine Sorte	JTI	AT	Tivoli	BAT	NL
Dimitrino Botschafter	Von Eicken	DE	Memphis Red	JTI	AT	Tobacco House No7	Western Tobacco Corp.	BE
Ducados	Altadis/IT	ES	Merit Blu	PMI	IT	Trend	JTI	AT
Elixyr	Landewyk Tobacco	DE	MM	Bulgartabac	BG	Tschick Blau	Tschick Fabrik	AT
Email	Manufattura Italiana Tabacco	GR	Mohawk Red	Grand River Enterprises	DE	Ventil Gigante	Tabaqueira	PT
Ernte23	IT	AT	MS Bianche	BAT	IT	Viceroy Blue	BAT	CZ
Esportazione*	BAT	IT	Muratti Azure	PMI	IT	Victory Blue	BAT	BG
Eura	BAT	IT	Nil Regie	JTI	DE	Vivas	Gallaher Group - JTI	ES
Eva Secrets*	Bulgartabac	BG	Nobel	Altadis/IT	ES	Vogue	BAT	LT
Excite Red	Von Eicken	AT	OME	Karelia	AT	West Silver	IT	IT
F6	PMI	DE	Oranje	N/A	NL	Winston Blue	JTI	LT

* Cigarettes excluded from the PCA model of reference WDCF-cigarette group.

BAT = British American Tobacco, CTIEC = China Tobacco International Europe, IT = Imperial Tobacco Group, JTI = Japan Tobacco International, PMI = Philip Morris International, N/A = not available.

Table S2 Details on the flavoured tobacco products

Brand	Name/Flavour	Company	Type of product	Note
Cigarettes				
Black Devil	Gray - chocolate flavour	Heupink & Bloemen	cigarettes	
Black Devil	Black - special flavour	Heupink & Bloemen	cigarettes	
Black Devil	Cappuccino	Heupink & Bloemen	cigarettes	
Black Devil	Chocolate	Heupink & Bloemen	cigarettes	
Black Devil	Vanilla	Heupink & Bloemen	cigarettes	
Black Devil	Flavoured	Heupink & Bloemen	cigarettes	
Bohem	Mojito	KT&G Korea	cigarettes	natural lime, mint and rum flavour
Boxer	Menthol	Leonard Dingler Ltd	cigarettes	
Craven A	Menthol	British American Tobacco	cigarettes	
Craven A	Original menthol	Carreras of Picadilly	cigarettes	
Cuba Libre	Flavoured	Continental Tobacco Corp.	cigarettes	
Dimitrino	Springwater - vanilla	Grande Fabrique de Cigarettes Egyptiennes	cigarettes	
Djarum	Black cappuccino	PT Djarum	cigarettes	cappuccino flavored kretek cigarette*
Glamm	Green - menthol	Japan Tobacco International	cigarettes	
Glamour	Menthol	Japan Tobacco International	cigarettes	
Goldfield	Green - menthol	Grand River Enterprises GmbH	cigarettes	
Gudang Garam	Kretek*	PT.Gudang Garam Tbk.	cigarettes	kretek cigarette*
Karelia	Menthol	Karelia	cigarettes	
Lifa	Strawberry	LVIV tobacco factory	cigarettes	
Lifa	Menthol	LVIV tobacco factory	cigarettes	
Marlboro	Filter Black	PMI/PT HM Sampoerna Tbk.	cigarettes	kretek cigarette*
Mond	Cherry	-	cigarettes	
Nanjing	Menthol	China Tobacco Jiangsu Industrial Co. Ltd.	cigarettes	
Parliament	Blue - menthol	Philip Morris International	cigarettes	
Raison	French black	KT&G Korea	cigarettes	
Raison	Sun Presso	KT&G Korea	cigarettes	
Sweetie	Strawberry	Planta Tabak Berlin	cigarettes	
Vibes	Flavoured	Vibes Tobacco Company	cigarettes	
Vogue	Menthe - menthol	British American Tobacco	cigarettes	
Vogue	Menthol	British American Tobacco	cigarettes	
Cigarettes with flavour in capsule				
Bohem	Double apple mint	KT&G Korea	cigarettes	flavour in capsule
Camel	Activate purple mint	JT international	cigarettes	menthol cigarette with grape flavour in capsule

Esse	Berry pop	KT&G Korea	cigarettes	kretek cigarette* with berry mint flavour in capsule
Esse	Change - applemint	KT&G Korea	cigarettes	kretek cigarette* with apple mint flavour in capsule
Esse	Change - grape	KT&G Korea	cigarettes	kretek cigarette* with minty grape flavour in capsule
Esse	Honey pop - caramel	KT&G Korea	cigarettes	flavour in capsule
Marlboro	Purple Burst	PMI	cigarettes	2 capsules - flavour ball & fresh ball
Raison	Ice Presso	KT&G Korea	cigarettes	coffee taste with apple mint-fresh flavour in capsule
Sampoerna	A mild menthol burst	Sampoerna Tbk.	cigarettes	kretek cigarette* with a menthol capusle
This	Africa Rula - amarula flavour	KT&G Korea	cigarettes	amarula flavour in capsule
This	Africa Mola - random five	KT&G Korea	cigarettes	five flavours in capsule (rula, apple, lime, orange, strawberry)
This	Plus - vanilla	KT&G Korea	cigarettes	vanilla flavour in capsule
Roll-your-own (RYO) tobacco				
7 Seas Mac Baren	Cherry blend	Mac Baren Tobacco	RYO tobacco	
CGW	Kretek*	Est. CGW 2010	RYO tobacco	
CGW	Vanilla	Est. CGW 2010	RYO tobacco	
CGW	Cappuccino	Est. CGW 2010	RYO tobacco	
CGW	Tiramisu	Est. CGW 2010	RYO tobacco	
CGW	Strawberry milkshake	Est. CGW 2010	RYO tobacco	
CGW	Blueberry mint	Est. CGW 2010	RYO tobacco	
CGW	Strawberry	Est. CGW 2010	RYO tobacco	
CGW	Special	Est. CGW 2010	RYO tobacco	
CGW	Banana milkshake	Est. CGW 2010	RYO tobacco	
Harvest	Double vanilla	Von Eicken Germany	RYO tobacco	tobacco blended with double vanilla flavour
Harvest	Cherry	Von Eicken Germany	RYO tobacco	tobacco blended with cherry flavour
Mac Baren	Jamaican coffee choice	Mac Baren Tobacco	RYO tobacco	tobacco with a flavour of coffee
Mac Baren	Vanilla choice	Mac Baren Tobacco	RYO tobacco	tobacco with a sweet vanilla flavour
Mac Baren	Double cherry choice	Mac Baren Tobacco	RYO tobacco	tobacco with a sweet cherry flavour
Mac Baren	Original choice	Mac Baren Tobacco	RYO tobacco	tobacco with a sweet vanilla and caramel flavour
Mac Baren	Apple choice	Mac Baren Tobacco	RYO tobacco	
Mac Baren	Vanilla cream	Mac Baren Tobacco	loose cut	flavouring cream & vanilla

*Kretek are cigarettes made with a blend of tobacco and ground clove buds.

Table S3 List of the compounds included in the in-house flavour database used for the evaluation of the tobacco products

No.	Compound Name	Formula	Mass	RT (min)	CAS No.	LRI	LRI (lit)	m/z
1	Butanoic acid	C4H8O2	88.05	5.37	107-92-6	797	793	**60**, 73, 55
2	Ethyl butyrate	C6H12O2	116.08	5.51	105-54-4	804	805	**71**, 73, 88
3	Butyl acetate	C6H12O2	116.08	5.88	123-86-4	817	814	**56**, 61, 55
4	Ethyl lactate	C5H10O3	118.06	5.88	97-64-3	818	813	**45**, 75, 47
5	Pyrazine, methyl-	C5H6N2	94.05	6.04	109-08-0	824	825	**94**, 67, 53
6	Furfural	C5H4O2	96.02	6.26	98-01-1	832	835	**95**, 96, 67
7	Butanoic acid, 3-methyl-	C5H10O2	102.07	6.57	503-74-2	844	840	**60**, 87, 45
8	3-Hexen-1-ol	C6H12O	100.09	6.72	544-12-7	849	851	**82**, 67, 55
9	Ethyl 2-methylbutyrate	C7H14O2	130.10	6.72	7452-79-1	849	850	**57**, 102, 74
10	Ethyl isovalerate	C7H14O2	130.10	6.80	108-64-5	852	853	**57**, 85, 88
11	2-Furanmethanol	C5H6O2	98.04	6.88	98-00-0	855	856	**98**, 97, 81
12	1-Hexanol	C6H14O	102.10	7.03	111-27-3	861	862	**56**, 69, 55
13	Isoamyl acetate	C7H14O2	130.10	7.43	123-92-2	877	876	**55**, 70, 61
14	Pyrazine, 2,5-dimethyl-	C6H8N2	108.07	8.43	123-32-0	912	911	**108**, 109, 81
15	Pyrazine, 2,3-dimethyl-	C6H8N2	108.07	8.68	5910-89-4	919	919	**108**, 67, 109
16	γ-Valerolactone	C5H8O2	100.05	9.64	108-29-2	949	950	**85**, 56, 57
17	Ethylacetoacetate	C6H10O3	130.06	9.67	141-97-9	949	954	**88**, 60, 85
18	Benzaldehyde	C7H6O	106.04	10.01	100-52-7	960	961	**106**, 105, 77
19	5-Methylfurfural	C6H6O2	110.04	10.14	620-02-0	964	962	**110**, 109, 53
20	Pyrazine, 2-methoxy-3-methyl-	C6H8N2O	124.06	10.24	2847-30-5	967	954	**124**, 109, 123
21	Hexanoic acid	C6H12O2	116.08	10.74	142-62-1	982	981	**60**, 73, 87
22	5-Hepten-2-one, 6-methyl-	C8H14O	126.10	10.94	110-93-0	988	987	**108**, 111, 55
23	(-)-ß-Pinene	C10H16	136.13	11.06	18172-67-3	992	990	**93**, 91, 69
24	3-Octanol	C8H18O	130.14	11.21	589-98-0	997	996	**59**, 55, 83
25	α-Phellandrene	C10H16	136.13	11.31	99-83-2	999	1001	**93**, 91, 92
26	Ethyl caproate	C8H16O2	144.12	11.37	123-66-0	1001	1001	**88**, 99, 60
27	Pyrazine, trimethyl-	C7H10N2	122.08	11.42	14667-55-1	1003	1005	**122**, 81, 123
28	Hexyl acetate	C8H16O2	144.12	11.86	142-92-7	1015	1014	**56**, 84, 69
29	p-Methylanisole	C8H10O	122.07	11.87	104-93-8	1015	1019	**122**, 121, 107
30	Acetylpyrazine	C6H6N2O	122.05	12.07	22047-25-2	1021	1023	**122**, 94, 80
31	o-Cymene	C10H14	134.11	12.19	527-84-4	1024	1023	**119**, 91, 134
32	D-Limonene	C10H16	136.13	12.32	5989-27-5	1028	1030	**107**, 93, 121
33	Cyclotene	C6H8O2	112.05	12.35	80-71-7	1029	1029	**112**, 69, 84
34	Eucalyptol	C10H18O	154.14	12.40	470-82-6	1030	1030	**139**, 84, 111
35	Benzylalcohol	C7H8O	108.06	12.56	100-51-6	1035	1032	**79**, 108, 107
36	Salicylaldehyde	C7H6O2	122.04	12.60	90-02-8	1036	1033	**122**, 121, 65
37	Benzeneacetaldehyde	C8H8O	120.06	12.86	122-78-1	1043	1045	**91**, 92, 65
38	γ-Caprolactone	C6H10O2	114.07	13.25	695-06-7	1054	1056	**85**, 56, 70
39	Isoamyl butyrate	C9H18O2	158.13	13.37	106-27-4	1058	1060	**70**, 89, 55
40	γ-Terpinene	C10H16	136.13	13.39	99-85-4	1058	1059	**93**, 91, 121
41	Acetophenone	C8H8O	120.06	13.42	98-86-2	1059	1062	**105**, 106, 120
42	Ethanone, 1-(1H-pyrrol-2-yl)-	C6H7NO	109.05	13.59	1072-83-9	1064	1063	**94**, 109, 66
43	Linalool oxide	C10H18O2	170.13	13.91	5989-33-3	1073	1072	**59**, 93, 94
44	Benzoyl formate	C8H8O2	136.05	13.91	104-57-4	1073	1076	**91**, 90, 108
45	Pyrazine, 2-ethyl-3,5-dimethyl-	C8H12N2	136.10	14.17	13925-07-0	1080	1080	**135**, 136, 108
46	Benzaldehyde, 4-methyl-	C8H8O	120.06	14.17	104-87-0	1080	1079	**119**, 91, 120
47	Pyrazine, tetramethyl-	C8H12N2	136.10	14.41	1124-11-4	1087	1087	**136**, 54, 137
48	Terpinolene	C10H16	136.13	14.46	586-62-9	1088	1088	**136**, 121, 93
49	Methyl benzoate	C8H8O2	136.05	14.49	93-58-3	1089	1092	**105**, 77, 136

50	o-Guaiacol	C7H8O2	124.05	14.52	90-05-1	1090	1089	**109**, 112, 67
51	Ethyl heptanoate	C9H18O2	158.13	14.64	106-30-9	1093	1096	**88**, 113, 115
52	iso-Amyl 2-methyl butyrate	C10H20O2	172.15	14.84	27625-35-0	1099	1102	**70**, 57, 85
53	Linalool	C10H18O	154.14	14.88	78-70-6	1100	1100	**71**, 93, 121
54	Nonanal	C9H18O	142.14	14.96	124-19-6	1102	1104	**57**, 70, 98
55	Isoamyl isovalerate	C10H20O2	172.15	15.12	659-70-1	1107	1105	**70**, 57, 85
56	Maltol	C6H6O3	126.03	15.43	118-71-8	1116	1114	**123**, 55, 71
57	Phenylethyl alcohol	C8H10O	122.07	15.48	60-12-8	1117	1118	**91**, 92, 65
58	2-Cyclopenten-1-one, 3-ethyl-2-hydroxy-	C7H10O2	126.07	15.63	21835-01-8	1121	1122	**126**, 83, 84
59	Methyl octanoate	C9H18O2	158.13	15.73	111-11-5	1124	1125	**74,** 87, 115
60	Isopulegol	C10H18O	154.14	16.50	89-79-2	1146	1146	**121**, 93, 84
61	p-Menthone	C10H18O	154.14	16.82	89-80-5	1154	1155	**139**, 112, 97
62	Benzyl acetate	C9H10O2	150.07	17.16	140-11-4	1164	1165	**108**, 107, 150
63	Isomenthol	C10H20O	156.15	17.19	23283-97-8	1165	1164	**71**, 81, 95
64	Terpinen-4-ol	C10H18O	154.14	17.40	562-74-3	1171	1171	**111**, 93, 71
65	Octanoic acid	C8H16O2	144.12	17.40	124-07-2	1171	1170	**60**, 73, 101
66	Ethyl benzoate	C9H10O2	150.07	17.40	93-89-0	1171	1170	**105**, 77, 122
67	DL-Menthol	C10H20O	156.15	17.43	15356-70-4	1172	1172	**81**, 95, 123
68	Methyl phenylacetate	C9H10O2	150.07	17.62	101-41-7	1177	1178	**91**, 150, 65
69	4-Methylacetophenone	C9H10O	134.07	17.87	122-00-9	1184	1183	**119**, 91, 65
70	p-Cymen-8-ol	C10H14O	150.10	17.93	1197-01-9	1186	1184	**135**, 91, 119
71	Hexanoic acid, butylester	C10H20O2	172.15	17.96	626-82-4	1186	1188	**117**, 99, 71
72	α-Terpineol	C10H18O	154.14	18.13	98-55-5	1191	1195	**121**, 93, 136
73	Hexyl butanoate	C10H20O2	172.15	18.15	2639-63-6	1192	1191	**71**, 89, 56
74	Methyl salicylate	C8H8O3	152.05	18.23	119-36-8	1194	1192	**120**, 92, 152
75	Ethyl maltol	C7H8O3	140.05	18.32	4940-11-8	1197	1199	**140**, 139, 97
76	Citronellol	C10H20O	156.15	19.44	1117-61-9	1229	1225	**69**, 81, 95
77	Pulegone	C10H16O	152.12	19.82	89-82-7	1240	1237	**152**, 81, 67
78	Carvone	C10H14O	150.10	19.97	99-49-0	1245	1244	**82**, 93, 108
79	Benzeneacetic acid, ethylester	C10H12O2	164.08	20.02	101-97-3	1246	1247	**91**, 164, 92
80	p-Anisaldehyde	C8H8O2	136.05	20.26	123-11-5	1253	1251	**135**, 136, 107
81	Piperitone	C10H16O	152.12	20.33	89-81-6	1255	1254	**82**, 110, 95
82	γ-Octalactone	C8H14O2	142.10	20.41	104-50-7	1257	1257	**85**, 86, 100
83	1,3-Dioxolane, 4-methyl-2-phenyl-	C10H12O2	164.08	20.72	2568-25-4	1266	1272	**163**, 105, 164
84	Cinnamaldehyde, (E)-	C9H8O	132.06	20.84	14371-10-9	1270	1270	**131**, 132, 103
85	Citral	C10H16O	152.12	20.85	5392-40-5	1270	1270	**69**, 84, 137
86	p-Ethylguaiacol	C9H12O2	152.08	21.15	2785-89-9	1279	1282	**137**, 152, 122
87	p-Anisyl alcohol	C8H10O2	138.07	21.22	105-13-5	1281	1282	**138**, 109, 137
88	Anethole	C10H12O	148.09	21.38	104-46-1	1286	1284	**148**, 147, 117
89	Menthyl acetate	C12H22O2	198.16	21.61	89-48-5	1293	1294	**95**, 81, 123
90	Carvacrol	C10H14O	150.10	21.92	499-75-2	1302	1298	**135**, 150, 91
91	Methyl decanoate	C11H22O2	186.16	22.65	110-42-9	1324	1324	**74**, 87, 143
92	Piperonal	C8H6O3	150.03	22.90	120-57-0	1332	1333	**149**, 150, 121
93	Methylanthranilate	C8H9NO2	151.06	23.22	134-20-3	1341	1343	**119**, 151, 120
94	Benzyl butyrate	C11H14O2	178.10	23.39	103-37-7	1347	1347	**108**, 91, 107
95	Triacetin	C9H14O6	218.08	23.57	102-76-1	1352	1350	**103**, 145, 116
96	4-Acetylanisole	C9H10O2	150.07	23.61	100-06-1	1353	1355	**135**, 77, 150
97	Eugenol	C10H12O2	164.08	23.82	97-53-0	1360	1359	**164**, 149, 165
98	γ-Nonalactone	C9H16O2	156.12	23.88	104-61-0	1362	1362	**85**, 114, 99
99	Copaene	C15H24	204.19	24.33	3856-25-5	1376	1376	**161**, 105, 119
100	Hydrocoumarin	C9H8O2	148.05	24.38	119-84-6	1377	1392	**148**, 120, 91
101	Cinnamic acid, methyl ester	C10H10O2	162.07	24.54	103-26-4	1382	1381	**131**, 103, 162
102	trans-ß-Damascenone	C13H18O	190.14	24.67	23726-93-4	1386	1385	**69**, 121, 105
103	cis-Jasmone	C11H16O	164.12	24.68	488-10-8	1386	1385	**122**, 149, 131
104	Hexyl hexanoate	C12H24O2	200.18	24.68	6378-65-0	1386	1385	**117**, 99, 56

105	Isobutyl phenylacetate	C12H16O2	192.12	24.83	102-13-6	1391	1392	**91**, 136, 137
106	Methyleugenol	C11H14O2	178.10	25.07	93-15-2	1398	1399	**178**, 147, 163
107	Vanillin	C8H8O3	152.05	25.09	121-33-5	1399	1400	**151**, 152, 123
108	2-Buten-1-one, 1-(2,6,6-trimethyl-1-cyclohexen-1-yl)-	C13H20O	192.15	25.61	35044-68-9	1416	1412	**177**, 192, 123
109	Caryophyllene	C15H24	204.19	25.64	87-44-5	1417	1418	**133**, 161, 105
110	p-Anisyl acetate	C10H12O3	180.08	25.71	104-21-2	1419	1419	**121**, 180, 120
111	α-Ionone	C13H20O	192.15	25.97	127-41-3	1427	1427	**121**, 93, 136
112	Cinnamyl acetate	C11H12O2	176.08	26.29	103-54-8	1438	1439	**115**, 133, 134
113	Humulene	C15H24	204.19	26.74	6753-98-6	1453	1454	**93**, 121, 147
114	trans-Geranylacetone	C13H22O	194.17	26.78	3796-70-1	1454	1453	**69**, 107, 151
115	Ethylvanillin	C9H10O3	166.06	26.79	121-32-4	1454	1453	**137**, 166, 138
116	Cinnamic acid, ethyl ester	C11H12O2	176.08	26.92	103-36-6	1458	1460	**131**, 148, 176
117	γ-Decalactone	C10H18O2	170.13	27.18	706-14-9	1467	1470	**85**, 69, 128
118	Methylvanillin	C9H10O3	166.06	27.63	120-14-9	1481	1478	**165**, 166, 77
119	Isoamyl phenylacetate	C13H18O2	206.13	27.75	102-19-2	1485	1490	**91**, 136, 70
120	ß-Ionone	C13H20O	192.15	27.82	14901-07-6	1488	1486	**177**, 178, 159
121	δ-Decalactone	C10H18O2	170.13	28.06	705-86-2	1496	1493	**99**, 71, 114
122	alpha-Farnesene	C15H24	204.19	28.27	502-61-4	1502	1503	**93**, 107, 119
123	Eugenol acetate	C12H14O3	206.09	29.03	93-28-7	1528	1526	**164**, 149, 131
124	2-Butanone, 4-(4-hydroxyphenyl)-	C10H12O2	164.08	29.51	5471-51-2	1545	1550	**107**, 94, 164
125	Coumarin, 6-methyl-	C10H8O2	160.05	29.91	92-48-8	1558	1564	**160**, 132, 131
126	γ-Undecalactone	C11H20O2	184.15	30.33	104-67-6	1573	1573	**85**, 55, 128
127	Benzophenone	C13H10O	182.07	31.86	119-61-9	1626	1625	**105**, 77, 182
128	α-Amylcinnamaldehyde	C14H18O	202.14	32.32	122-40-7	1643	1645	**129**, 201, 202
129	Methyl dihydrojasmonate	C13H22O3	226.16	32.48	24851-98-7	1648	1650	**83**, 153, 82
130	δ-Dodecalactone	C12H22O2	198.16	34.13	713-95-1	1708	1705	**99**, 71, 55
131	Isoamyl cinnamate	C14H18O2	218.13	34.88	7779-65-9	1736	1719	**131**, 147, 103
132	Benzyl Benzoate	C14H12O2	212.08	35.72	120-51-4	1768	1765	**105**, 194, 91
133	Benzylcinnamate	C16H14O2	238.10	43.67	103-41-3	2094	2096	**91**, 131, 192

RT – retention time;
LRI – calculated linear retention index;
LRI (lit) – linear retention index taken from databases PubChem® National Library of Medicine. https://pubchem.ncbi.nlm.nih.gov/ (accessed January 2020) and NIST Chemistry WebBook. https://webbook.nist.gov/chemistry/ (accessed January 2020);
m/z – m/z values used as quantifier (bold print) and qualifier ions.

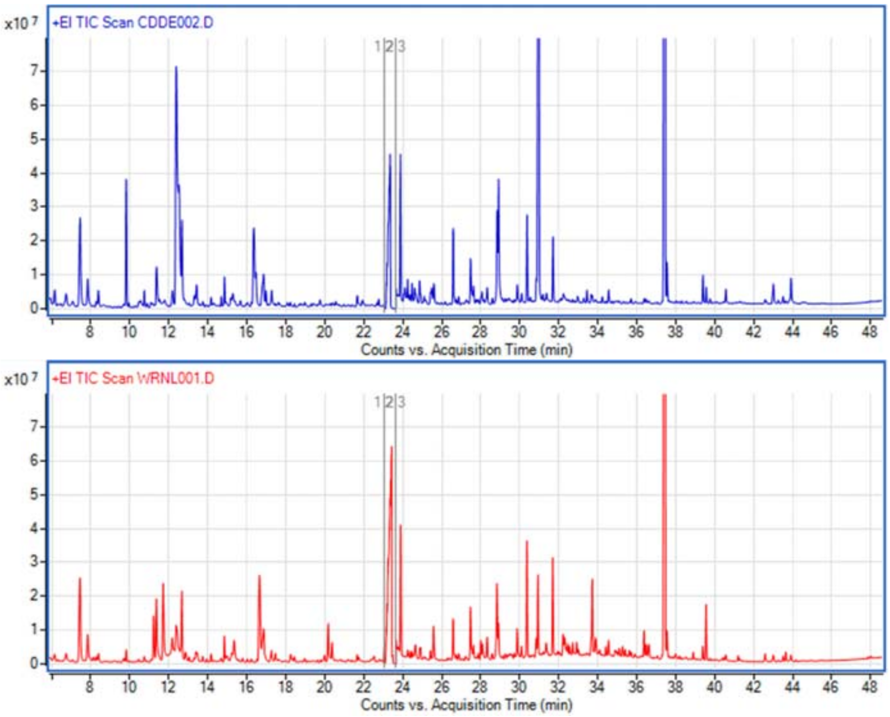

Fig. S1 Examples of total ion chromatograms of reference WDCF-cigarettes acquired by thermal desorption GC - QTOF MS

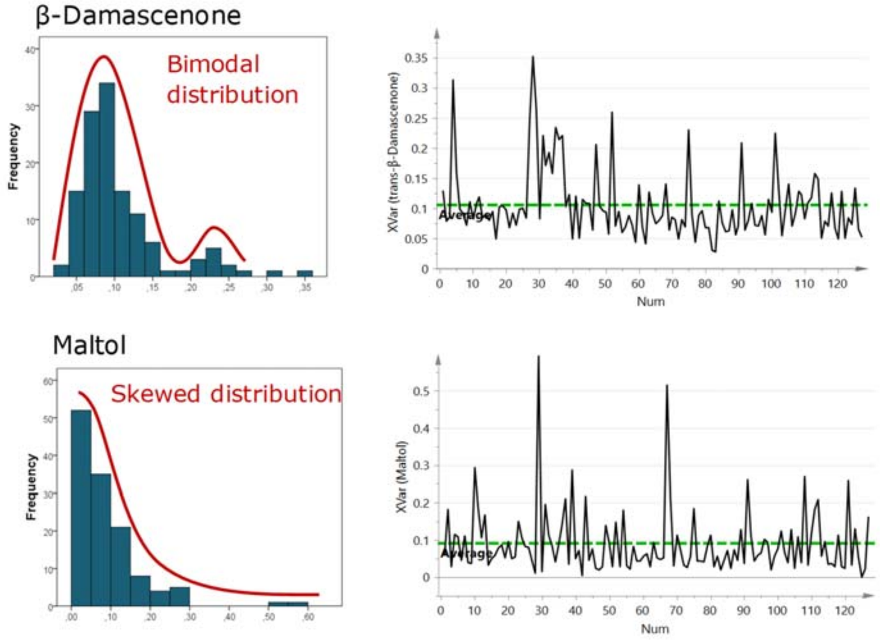

Fig. S2 Distribution patterns and relative responses of β-damascenone and maltol measured in reference group of non-flavoured cigarettes

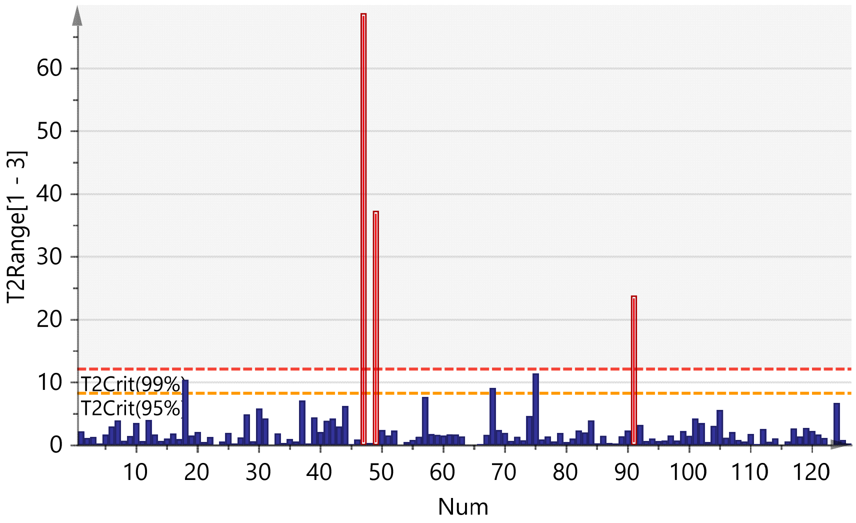

Fig. S3 Hotelling's T2 range plot for PCA model of entire data for reference group of WDCF-cigarettes. The Hotelling's T2 range plot displays the distance from the origin in the model plane (score space) for each observation. It is calculated as the sum over the selected range of components of the scores in square divided by their standard deviations in square. Values larger than the yellow limit (95% confidence region of the model) are suspect; values larger than the red limit (99% confidence region of the model) can be considered as outliers. Bars highlighted in red represents the three samples assumed as outliers, which were excluded from the final PCA model

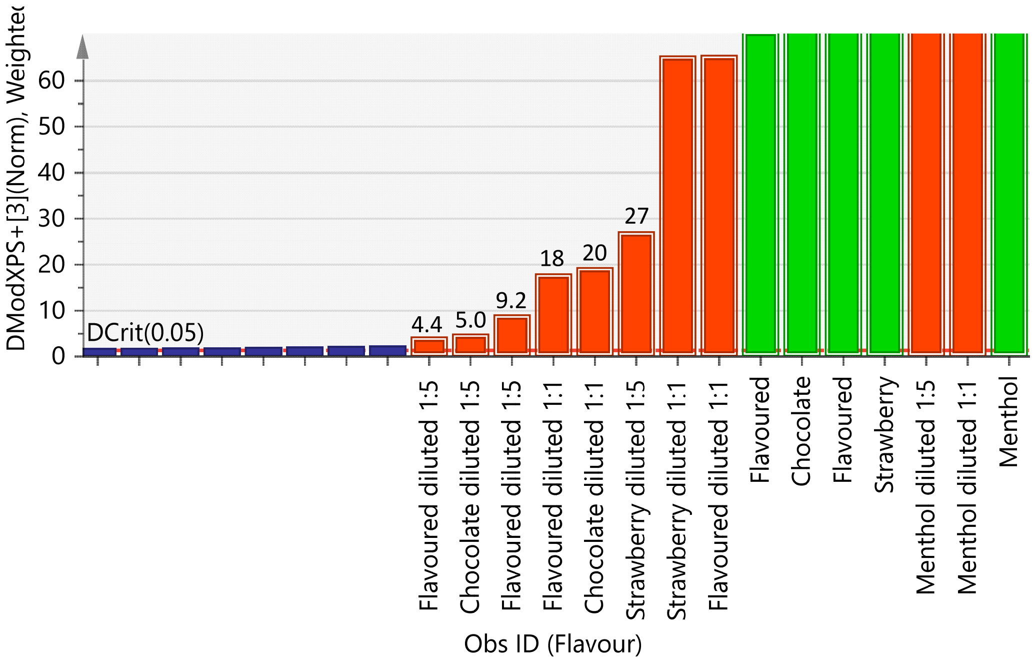

Fig. S4 DModX PS+ plot: Distance of selected flavoured cigarette samples original (green bars) and diluted in ratios of 1:1 and 1:5 (both orange bars) to the reference model. DModX PS+ is proportional to the residual standard deviation displayed in normalised unit, which is the absolute DModX divided by the pooled residual standard deviation of the model. DCrit, the critical value computed from the F-distribution for the model, at a significance level of 0.05, is equal to 1.18

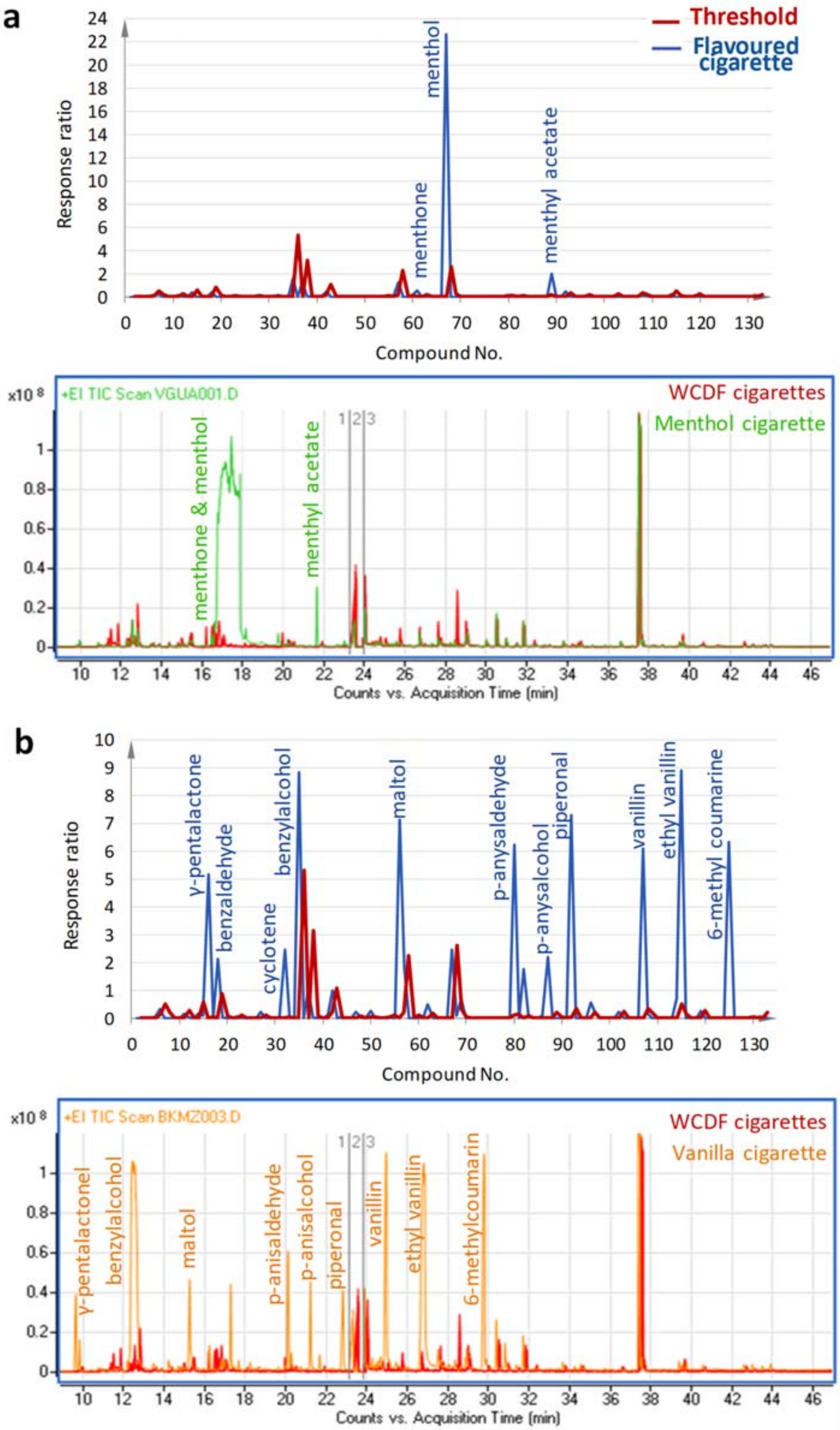
a
Threshold
Flavoured cigarette
Response ratio
Compound No.
menthol
menthone
menthyl acetate
WCDF cigarettes
Menthol cigarette
+EI TIC Scan VGUA001.D
x10 8
menthone & menthol
menthyl acetate
Counts vs. Acquisition Time (min)
b
Response ratio
Compound No.
γ-pentalactone
benzaldehyde
cyclotene
benzylalcohol
maltol
p-anysaldehyde
p-anysalcohol
piperonal
vanillin
ethyl vanillin
6-methyl coumarine
+EI TIC Scan BKMZ003.D
x10 8
WCDF cigarettes
Vanilla cigarette
γ-pentalactonel
benzylalcohol
maltol
p-anisaldehyde
p-anisalcohol
piperonal
vanillin
ethyl vanillin
6-methylcoumarin
Counts vs. Acquisition Time (min)

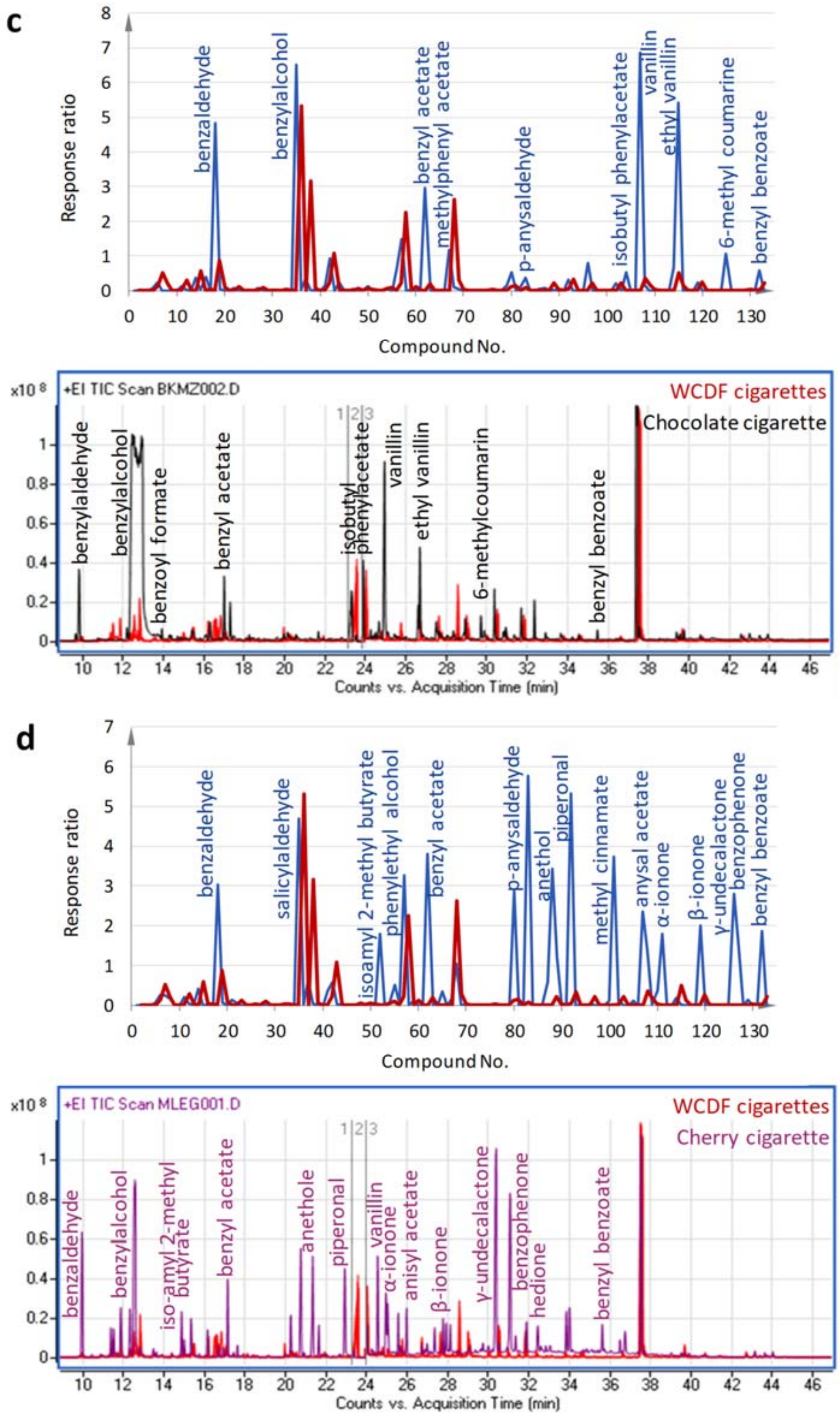

Fig. S5 Upper plots: overlaid profile of threshold values (red line) with the profile measured in flavoured cigarettes (blue line); lower plots: overlaid total ion chromatograms of six tobacco fillers from the group of reference WDCF-cigarettes (red line) and tobacco fillers of flavoured cigarettes **a**) menthol flavoured, **b**) vanilla flavoured, **c**) chocolate flavoured, **d**) cherry flavoured. Highly abundant flavour components are labelled.
